# Association of targeted multiplex PCR with resequencing microarray for the detection of multiple respiratory pathogens

**DOI:** 10.3389/fmicb.2015.00532

**Published:** 2015-05-28

**Authors:** Hongwei Shen, Bingqing Zhu, Shulian Wang, Haolian Mo, Ji Wang, Jin Li, Chen Zhang, Huashu Zeng, Li Guan, Weixian Shi, Yong Zhang, Xuejun Ma

**Affiliations:** ^1^Key Laboratory of Medical Virology, Ministry of Health, Chinese Center for Disease Control and Prevention, National Institute for Viral Disease Control and PreventionBeijing, China; ^2^Futian District Center for Disease Control and PreventionShenzhen, China; ^3^State Key Laboratory for Infectious Disease Prevention and Control, Chinese Center for Disease Control and Prevention, National Institute for Communicable Disease Control and PreventionBeijing, China; ^4^Third Hospital of Beijing Armed Police Corps LaboratoryBeijing, China; ^5^Beijing Center for Disease Control and Prevention, Institute for Infectious Disease and Endemic Disease ControlBeijing, China

**Keywords:** resequencing microarray, RPM-IVDC1, community-acquired pneumonia, multiple respiratory pathogens, detection

## Abstract

A large number of viral and bacterial organisms are responsible for community-acquired pneumonia (CAP) which contributes to substantial burden on health management. A new resequencing microarray (RPM-IVDC1) associated with targeted multiplex PCR was recently developed and validated for multiple respiratory viruses detection and discrimination. In this study, we evaluated the capability of RPM-IVDC1 for simultaneous identification of multiple viral and bacterial organisms. The nasopharyngeal aspirates (NPAs) of 110 consecutive CAP patients, aged from 1 month to 96 years old, were collected from five distinct general hospitals in Beijing during 1-year period. The samples were subjected to the RPM-IVDC1 established protocol as compared to a real-time PCR (qRT-PCR), which was used as standard. The results of virus detection were consistent with those previously described. A total of 37 of *Streptococcus pneumoniae*, 14 of *Haemophilus influenzae*, 10 of *Mycoplasma pneumoniae*, two of *Klebsiella pneumoniae* and one of *Moraxella catarrhalis* were detected by RPM-IVDC1. The sensitivities and specificities were compared with those of qRT-PCR for *S. pneumoniae* (100, 100%, respectively)*, H. influenzae* (92.3, 97.9%, respectively)*, M. pneumoniae* (69.2, 99.0%, respectively)*, K. pneumoniae* (100, 100%, respectively), and *M. catarrhalis* (100, 100%, respectively). Additional 22 of *Streptococcus* spp., 24 of *Haemophilus* spp. and 16 of *Neisseria* spp. were identified. In addition, methicillin-resistant and carbapenemases allele were also found in nine of *Staphylococcus* spp. and one of *K. pneumoniae*, respectively. These results demonstrated the capability of RPM-IVDC1 for simultaneous detection of broad-spectrum respiratory pathogens in complex backgrounds and the advantage of accessing to the actual sequences, showing great potential use of epidemic outbreak investigation. The detection results should be carefully interpreted when introducing this technique in the clinical diagnostics.

## Introduction

Acute respiratory infection (ARI) is a leading cause of morbidity and mortality worldwide. The community-acquired pneumonia (CAP) resulted from ARI remains to be a common and serious illness and contributes to a substantial burden on health services (Bartlett and Mundy, 1995; Welte et al., 2012). A large and growing number of viral and bacterial organisms are responsible for CAP (Juven et al., 2000; Johansson et al., 2010). In addition to the predominant causative agents (most commonly *Streptococcus pneumoniae, Haemophilus influenzae, Mycoplasma pneumoniae*, respiratory syncytial virus, and influenza virus) (Rudan et al., 2008, 2013), new pathogens including Coronaviruses (CoV-NL63 and CoV-HKU1) (Moes et al., 2005; Woo et al., 2005), human metapneumovirus (hPMV) (Hamelin et al., 2005), human bocavirus (hBoV) (Fry et al., 2007) and H7N9 (Uyeki and Cox, 2013) are frequently being reported. Some of these pathogens can cause severe cases of pneumonia and pandemic (Uyeki and Cox, 2013).

A large number of studies have been carried out to detect the etiology of CAP, showing that high prevalence of mixed viral-bacterial infections occured in CAP patients (Tsolia et al., 2004; Kumar et al., 2008). In addition, some bacterial infections were associated with critically illness (Trouillet et al., 1998; Gruson et al., 2000). Because of the difficulty in differentiating between pathogens based on clinical signs or radiology, the identification of pathogens in severe respiratory tract infections would be of great value to assist medical treatment. Therefore, to improve the capability of diagnosis of serious respiratory illnesses a powerful assay that is capable of broad-spectrum pathogen detection and identification is urgently needed.

The resequencing microarray, depending on hybridization between probes attached to the array and complementary fragments of nucleic acids in the specimens, offered detailed microbial characterization and broad-range pathogen detection (Leski et al., 2012). An important feature of this approach was that it generated actual sequences of the detected targets. The target sequence generated by the hybridization between closely overlapping probe sets and complementary fragments was compared with a sequence database. Benefit from the advantage of obtaining reliable sequence information, the microarray-based resequencing assay achieved high-resolution target discrimination and identification of target mixtures (Leski et al., 2012). Thus, this technology has been applied for high-resolution multiplexed diagnostics for clinical syndromes with similar symptoms (Lin et al., 2006, 2007; Leski et al., 2009).

We previously described a new resequencing pathogen microarray based assay (RPM-IVDC1) showing excellent capability for detecting broad-range CAP-associated viruses (Shen et al., 2013). The RPM-IVDC1 assay could sequence 47,974 bp of both strands of targeted gene sequences, distributed across 183 detector tiles (each 224-bp in length), representing 86 types/subtypes of viral pathogens and 21 other respiratory tract pathogens that cause respiratory infection (Shen et al., 2013). But the capacity of RPM-IVDC1 for simultaneous detection of viral and bacterial pathogens with improved application in microbial diagnostics has not been validated. In this study, 110 Nasopharynx aspirates (NPAs) from CAP patients in five district hospitals in Beijing were selected and tested by RPM-IVDC1 assay. The bacterial detection results were additionally confirmed by specific real-time PCR assays which is the gold standard in this case.

## Materials and methods

### Samples

From January 2011 to December 2011, participants for radiologically verified CAP were enrolled from Beijing Pneumonia Surveillance. The data on all hospital admissions, other important medical events, culture results of mycoplasma and viral infections were collected. Among these participants, the patients with complete information regarding demographic character, radiological results and clinical manifestations were enrolled in this study. A total of 110 CAP patients (62 of whom were female) meeting the criteria, aged from 1 month to 96 years, were selected in this study (Table 1).

**Table 1:** **The epidemiological characteristics of samples (*n* = 110) in this study**.

**Category**	**Subcatergory**	**No. (%)**
Sex	Female	62 (56.4)
	Male	48 (43.6)
Age (years)	0–4	21 (19.1)
	5–14	19 (17.3)
	15–24	20 (18.2)
	25–59	25 (22.7)
	60–96	25 (22.7)

### Specimen collection and processing

NPAs were collected from all patients admitting hospitals. Five milliliters of sterile saline was squirted into one nostril, and a 6Fr suction catheter was placed into the nasopharynx using the opposite nostril, pointing the end of the tube directly backward, with the patient aspirating while the suction catheter was gently removed. After ensuring that the specimen was cloudy and had mucus and cells, it was immediately placed on ice and mixed with a universal transport medium (UTM, Copan Diagnostic, Inc., Murrieta, CA) before transporting to ultra-low temperature freezer in district hospital. Next, batches of specimens were transported to Beijing CDC in 5 days, on dry ice, where they were divided into aliquots for culture and PCR, and the remainder was stored at −80°C.

### Nucleic acid extraction and PCR

The complete nucleic acid of microorganisms and human cells from NPAs was extracted and purified using the MasterPure Complete DNA and RNA Purification kit (Epicenter Technologies, Madison, WI) according to the manufacture's recommended protocols. Purified DNA and RNA were frozen at −80°C in 15 μl aliquots for the following PCR. A reverse transcription PCR using SuperScript III (Invitrogen Life Technologies, Carlsbad, CA) associated with five targeted multiplex PCR assays (assays A-E) were performed before subjecting to RPM-IVDC1 process. Each of the multiplex PCR tubes contained primers representing different pathogens and internal controls as previously described (Shen et al., 2013). Primer mixes A-D were previously described (Shen et al., 2013) (see Supplementary Tables 1–4 in the Supplemental Material), while primer mix E contained 37 primer pairs corresponding to 35 target genes of *Bordetella pertussis, Chlamydia trachomatis, Chlamydophila pneumoniae, Chlamydophila psittaci, Corynebacterium diphtheriae, H. influenzae, K. pneumoniae, Legionella pneumophila, Moraxella catarrhalis, M. pneumoniae, Pseudomonas aeruginosa, Pseudomonas* spp., *Staphylococcus aureus, S. pneumoniae, Sreptococcus agalactiae, Streptococcus pyogenes* (group A *Streptococcu*s, GAS), *Francisella tularensis, Neisseria meningitides, Neisseria gonorrhoeae, Neisseria* spp. and one internal control (TIM-2) (see Supplementary Table 5 in the Supplemental Material). The amplification reactions were carried out in a Px2 Thermo Cycler (Thermo Electron Corp, Vantaa, Finland) with the same reaction conditions as previously described (Shen et al., 2013).

### RPM-IVDC1 processing

The products from five multiplex PCR assays were pooled together and subjected to purification (Qiagen, Germany), processing, and hybridization in a GeneChip Hybridization Oven 640 (Affymetrix Inc, Santa Clara, CA) according to the manufacture's recommended protocol(Malanoski et al., 2006; Lin et al., 2007). However, the hybridization was performed at 49°C for 12 h, which was much shorter than the time previously reported resequencing microarray adopted(Lin et al., 2007, 2009). The process of staining and scanning followed by hybridization were performed in a GeneChip Fluidics Station 450 (Affymetrix Inc, Santa Clara, CA). The data collected by microarray were transferred to a computer to create scanned images. A FASTA output file produced by processing the scanned image using TessArray Sequence Analysis (TSEQ) (Malanoski et al., 2006) was subjected to alignment with corresponding detector tile sequences. The alignment results were evaluated by “C3 Score,” which means the total number of TSEQ-identified nucleotides that appear in runs of three or more consecutive (non-N) base calls, expressed as percentage of the length (nucleotides) of each RPM-IVDC1 detector tile sequence (Lin et al., 2009).

### Assessment of RPM-IVDC1 results

Detection and quantification of the targets identified by RPM-IVDC1 were confirmed by using qRT-PCR (7500 real-time PCR system; Applied Biosystems) as described previously. These targets included *S. pneumoniae, H. influenzae, M. catarrhalis, K. pneumoniae*, and *M. pneumoniae* (Table 2). Compared with the targets identified by RPM-IVDC1, these qRT-PCRs targeted other regions of the same gene or other genes different from RPM-IVDC1 targets. A positive and negative control in each set PCR assay was included to survey the possibility of laboratory contamination. All the qRT-PCR methods were reported previously (Table 2) and validated in the lab of National Institute for Communicable Disease Control and Prevention, Chinese Center for Disease Control and Prevention. For culture of *M. pneumoniae*, a 50 μl aliquot of NPAs was plated on SP4 agar and the putative *M. pneumoniae* colonies were verified by nested PCR (Dorigo-Zetsma et al., 1999).

**Table 2:** **Additional oligonucleotide primers and probes used in the qRT-PCR**.

**Organisms**	**Gene target^a^**	**Primer sequence (5′-3′)^b^**	**Probe (5′-3′)**	**Citation**
*S. pneumoniae*	*ply*	F: AGCGATAGCTTTCTCCAAGTGG R: CTTAGCCAACAAATCGTTTACCG	ACCCCAGCAATTCAAGTGTTCGCG	Greiner et al., 2001
*M. pneumoniae*	*Cards*	F: TTTGGTAGCTGGTTACGGGAAT R: GGTCGGCACGAATTTCATATAAG	TGTACCAGAGCACCCCAGAAGGGCT	Winchell et al., 2008
*H. influenzae*	*P6*	F: ACTTTTGGCGGTTACTCTG R: TGTGCCTAATTTACCAGCAT	GCATATTTAAATGCAACACCAGCTGCT	van Ketel et al., 1990
*K. pneumoniae*	KPCs	F: TCTGGACCGCTGGGAGCTGG R: TGCCCGTTGACGCCCAATCC	CGCGCGCCGTGACGGAAAGC	Cole et al., 2009
*M. catarrhalis*	*copB*	F: GTGAGTGCCGCTTTACAACC R: TGTATCGCCTGCCAAGACAA	TGCTTTTGCAGCTGTTAGCCAGCCTAA	Greiner et al., 2003
*S. aureus*	*femA*	F: TGCTGGTGGTACATCAAA R: ACGGTCAATGCCATGATTTAA	ATTTTGCCGGAAGTTATGCAGTGCAATG	Ruimy et al., 2008
*P. aeruginosa*	*oprL*	F: CGAGTACAACATGGCTCTGG R: ACCGGACGCTCTTTACCATA	CCT GCA GCA CCA GGT AGC GC	Feizabadi et al., 2010
*M. tuberculosis^c^*	IS*6110*	F: GAACGGCTGATGACCAAACT R: ATCAGCGATCGTGGTCCTG	/	Luo et al., 2010
*N. meningitides*	*ctrA*	F: GTGATGGTGCGTTTGGTGCAGAATA R: CACATTTGCCGTTGAACCACCTACC	CAACACACGCTCACCGGCTGCCGTCAGCGGCATAC	Boving et al., 2009
GAS^d^	*speB*	F:GTCAACATGCAGCTACAGGA R:AATACCAACATCAGCATCA	/	Louie et al., 1998

a *ply, pneuomolysin; Cards, CARDS toxin; P6, outer membrane protein P6; KPCs, beta-lactamase KPC-2; copB, outer membrane protein; femA, factor essential for methicillin resistance; oprL, peptidoglycan-associated lipoprotein; IS6110, IS6110 hypothetical protein; ctrA, capsular transport protein; speB, pyrogenic exotoxin B*.

b *F, forward; R, reverse*.

c *The confirmative assay for M. tuberculosis was based on the melting curve of the real-time PCR*.

d *The confirmative assay for M. tuberculosis was PCR followed by sequencing*.

For others potential respiratory bacteria including *S. aureus, P. aeruginosa, Mycobacterium tuberculosis, Neisseria meningitides*, and GAS not covered by RPM-IVDC1, an additional set of qRT-PCRs targeting these organisms were performed in consideration of probability of false-negative results. All the qRT-PCR methods were reported previously (Table 2) and validated in our laboratory.

### Statistical analysis

Eligibility and classification of the clinical syndromes of CAP were determined from the original record of each item on the medical history and examination in the database. RPM-IVDC1-identified results were compared with qRT-PCRs to evaluate its sensitivity, specificity, and overall diagnostic accuracy. The frequency distributions of the important pathogens among different ages of CAP patients and among different seasons were described and compared with those reported in the literatures. The C3 score, expressed as percentage of the length (nucleotides) of each RPM-IVDC1 detector tile sequence, was set to 40 in this study. The alignment results of targeted bacterial sequences with C3 score above 40 were tested as positive (Table 3).

**Table 3:** **Bacterial organisms detected in 110 (age, 0.1–96 years) CAP patients by RPM-IVDC1**.

**Organisms**	**No. of samples positive**	**Targeted gene^a^ (average of C3 score)**
*Streptococcus* spp.	59	*gyrA* (73.4)
*S. pneumoniae*	37^b^	*ply* (92.3)^c^
*Staphylococcus* spp.	10	*mecA* (95.1)
*Haemophilus* spp.	38	*skp* (84.5), *adk* (86.4)
*H. influenzae*	14^d^	*skp* (92.4),*adk* (91.8)
*M. pneumoniae*	10	*adhP1* (84.75), *cards* (97.1)
*K. pneumoniae*	2	*fumC* (71)
*M. catarrhalis*	1	*CopB* (77)
*Neisseria* spp.	16	*gyrA* (40.3)

a *gyrA, gyrase A subunit; ply, pneumolysin; mecA, penicillin binding protein, skp, outer membrane protein 26; adk, adenylate kinase; adhP1, P1 adhensin; cards, cards toxin; fumC, fumarase C; copB, outer membrane protein B2*.

b *The remaining of 22 Streptococcus spp.-positive samples were identified as non-S. pneumoniae by sequence alignment*.

c *A total of two samples of which the C3 scores were above 88 were identified as Streptococcus pseudopneumoniae, the alignment results were confirmed by specific qRT-PCR (Wessels et al., 2012)*.

d *The remaining of 24 Haemophilus spp.-positive samples were identified as non- H. influenzae by sequence alignment*.

### Ethical approval

All aspects of this study were performed in accordance with national ethics regulations and approved by the Institutional Review Boards of the Center for Disease Control and Prevention of China and the Ethics Committee of the Beijing Center for Disease Control and Prevention (Beijing CDC). The participants received written information regarding the purpose of the study and of their right to confidentiality. Individual written informed consent was obtained from adult participants or children's parents or guardians.

## Results

### Simultaneous detection of viral and bacterial microorganisms

The simultaneous detection results of viral pathogens were the same as previously described (Shen et al., 2013), demonstrating that the high specificity of RPM-IVDC1 for viruses detection was not interfered by the complex background. A viral etiology was found in 53 patients (48%), 12 of whom had more than one viral species identified. It was noted that the housekeeping genes were targeted and the corresponding bacteria could only be identified at species-level (e.g., gyrase A subunit gene of *Neisseria* spp.).

*M. pneumoniae* (8/10) and *S. pneumoniae* (21/37) were mainly detected in virus-negative samples, indicating that these organisms may be the causative agent of CAP. *S. pneumoniae* (4/4) and *H. influenzae* (3/4) were frequently found in the coronavirus (CoV)-infected samples, which may be the result of secondary infection induced by CoV or co-infection. A similar situation occurred in parainfluenza virus (PIV) and respiratory syncytial virus (RSV) infections, where four of seven PIV-positive and three of five RSV-positive samples were positive for *S. pneumoniae*, compared with detection rate of 24% (6/25) for *S. pneumoniae* among others virus infections (data not shown).

### Clinical sensitivity, specificity, and positive predictive value (PPV)

The chosen cutoff value resulted in 37 samples positive for *S. pneumoniae*, 14 positive for *H. influenzae*, 10 positive for *M. pneumoniae*, one positive for *M. catarrhalis*, and two positive for *K. pneumoniae*. Additional 22 specimens were positive for *Streptococcus* spp., 24 were positive for *Haemophilus* spp., 16 were positive for *Neisseria* spp., and 10 were positive for *Staphylococcus* spp. *_mecA_* (Table 3). According to the results of confirmatory qRT-PCR, two of 14 *H. influenzae* microarray-positive specimens were false positive. The RPM-IVDC1 assay failed to identify 4 specimens that were positive for *M. pneumoniae* by qRT-PCR. One NPAs sample with a true-positive result for *M. pneumoniae*, as confirmed by mycoplasma culture, was not detected by qRT-PCR (Table 4). The specimens that were positive for *Streptococcus* spp., *Haemophilus* spp., and *Neisseria* spp. were confirmed by 16s rRNA sequencing(Klindworth et al., 2013) (data not shown). Of these, nine of 10 *Staphylococcus* spp. *_mecA_* positive samples were confirmed by qRT-PCR and one was false positive (Table 5).

**Table 4:** **Comparison of RPM-IVDC1 with specific qRT-PCR assays for detection of *M. pneumoniae, S. pneumoniae*, and *H. influenzae*^a^**.

***M. pneumoniae***			***S. pneumoniae***			***H. influenzae***		
**No. of samples positive^b^**		**Total no. of patient samples with a positive result**	**No. of samples positive**		**Total no. of patient samples with a positive result**	**No. of samples positive**		**Total no. of patient samples with a positive result**
**RPM-IVDC1**	**qRT-PCR**		**RPM-IVDC1**	**qRT-PCR**		**RPM-IVDC1**	**qRT-PCR**	
+	+	9	+	+	37^e^	+	+	12^f^
+	−	1^c^	+	−	0	+	−	2^g^
−	+	4^d^	−	+	0	−	+	1
10	13	14	37	37	37	14	13	15

a *M. pneumoniae, Mycoplasma pneumonia; S. pneumonia, Streptococcus pneumonia; H. influenzae, Haemophilus influenzae*.

a *+ and −, samples positive or negative by the analysis applied*.

c *The result was confirmed by mycoplasma culture*.

d *The CT values of the four false negative of RPM-IVDC1 assay for M. pneumoniae detection were 35, 36, 37, and 37*.

e *The other 22 Streptococcus spp.-positive samples were not detected by S. pneumonia- specific qRT-PCR*.

f *The other 24 Haemophilus spp.-positive samples were not detected by H. influenzae-specific qRT-PCR*.

g *The retesting results of the two specimens false positive for H. influenzae were in concordance with the reference method*.

**Table 5:** **Comparison of RPM-IVDC1 with specific qRT-PCR assays for detection of other respiratory bacteria**.

**Organism**	**No. of samples positive**		**Total no. of patient samples with a positive result**
	**RPM-IVDC1**	**qRT-PCR**	
*Staphylococcus* spp.	10^a^	9	10
*S. aureus*	0	0	0
*K. pneumoniae*	2	2	2
*M. catarrhalis*	1	2	2
*Neisseria* spp.	16	ND^b^	16
*N. meningitides*	0	0	0
*P. aeruginosa*	0	0	0
*M. tuberculosis*	0	0	0
GAS	0	0	0

a *A total of nine positive samples for mecA allele were confirmed by qRT-PCR, the other one false positive sample was retested as mecA allele negative*.

b *ND, not detected by qRT-PCR. However, the results were confirmed by 16s rRNA sequencing (Klindworth et al., 2013)*.

The sensitivities, specificities, and PPVs for the RPM-IVDC1 assay were shown in Table 6. The sensitivities for the three most important bacterial pathogens, *S. pneumoniae, H. influenzae*, and *M. pneumoniae*, were 100, 92.3, and 69.2%, respectively. The number of specimens positive for *K. pneumoniae* and *M. catarrhalis* was too small to allow for a realistic evaluation of sensitivities. The resequencing assay showed excellent specificities for these organisms, ranging from 97.9 to 100%. The PPVs for the five common pathogens varied from 85.7 to 100%.

**Table 6:** **Sensitivity, specificity, and PPV for the RPM-IVDC1**.

**Organism**	**Sensitivity**		**Specificity**		**PPV**	
	**%**	**No. of samples positive/total no. tested**	**%**	**No. of samples negative/total no. tested**	**%**	**No. of samples positive/total no. tested**
*S. pneumoniae*	100	37/37	100	73/73	100	37/37
*H. influenzae*	92.3	12/13	97.9	95/97	85.7	12/14
*M. pneumoniae*	69.2	9/13	99.0	96/97	90.0	9/10
*K. pneumoniae*	(100)^a^	2/2	100	108/108	(100)	2/2
*M. catarrhalis*	(100)	1/1	100	109/109	(100)	1/1

a *The numbers in parentheses were based on numbers of samples too small to perform a valid calculation*.

### The distributions of the respiratory pathogens among the CAP patients

Due to the commensal and asymptomatic colonization of the upper respiratory tract, it was difficult to distinguish colonization from pathogen infection. Another hurdle for determining the etiology of these CAP patients was that the RPM-IVDC1 could only identify some organisms at species-level by conserved targeting (e.g., *Neisseria* spp.). Therefore, the distributions of only the four most important pathogens were analyzed (Table 7). The detection rate of *S. pneumoniae* and *H. influenzae* in autumn (September–November) was 41.4 and 17.2%, compared with 30.9 and 8.6% of the other seasons, respectively. Among the children aged less than 5 years, *S. pneumoniae* and *H. influenzae* were detected in 68.2 and 22.7% of this age group, compared with 25 (22/88) and 8.0% (7/88) of the other groups, respectively. The positive results for *M. pneumoniae* were mainly found in age group of 5 to 14 years (26.3%, 5/19) and of 15 to 24 years (15.0%, 3/20). Interestingly, the detection rate of *M. pneumoniae* among the male (14.6%) was obviously higher than the female patients (4.8%). The two positive results for *K. pneumoniae* were detected from two male patients aged 72 and 53 years, respectively.

**Table 7:** **The distribution of four important respiratory bacteria among 110 CAP patients (age, 0.1–96 years) detected by RPM-IVDC1**.

**Organism**	**No. of samples positive/total no. tested(%)**										
	**Sex**		**Age (years)**					**Month**			
	Male	Female	0 ~ 4	5 ~ 14	15 ~ 24	25 ~ 59	60 ~ 96	3 ~ 5	6 ~ 8	9 ~ 11	12 ~ 2
*S. pneumoniae*	31.3 (15/48)	35.5 (22/62)	66.7 (14/21)	36.8 (7/19)	25.0 (5/20)	28.0 (7/25)	16.0 (4/25)	20.8 (5/24)	34.8 (8/23)	41.4 (12/29)	35.3 (12/34)
*H. influenzae^a^*	10.4 (5/48)	11.3 (7/62)	14.3 (3/21)	5.3 (1/19)	5.0 (1/20)	12.0 (3/25)	16.0 (4/25)	8.3 (2/24)	8.7 (2/23)	17.2 (5/29)	8.8 (3/34)
*M. pneumoniae*	14.6 (7/48)	4.8 (3/62)	4.8 (1/21)	26.3 (5/19)	15.0 (3/20)	4.0 (1/25)	0 (0/25)	12.5 (3/24)	4.3 (1/23)	6.9 (2/29)	11.8 (4/34)
*K. pneumoniae*	0 (0/48)	3.2 (2/62)	0 (0/21)	0 (0/19)	0 (0/20)	4.0 (1/25)	4.0 (1/25)	4.2 (1/24)	0 (0/23)	0 (0/29)	2.9 (1/34)

a *The two specimens with false-positive results for H. influenzae were not included*.

## Discussions

The simultaneous detection of broad-spectrum pathogens causing similar clinical syndromes is beneficial to public health management. The previously reported RPM-IVDC1 assay offered a sensitive, accurate, and specific identification for multiple respiratory tract viruses infection (Shen et al., 2013). However, the ability of this assay to diagnose bacterial infection was not validated. Therefore, the purpose of this study is to evaluate the use of RPM-IVDC1 assay for detecting both respiratory viruses and bacteria using the same batch of NPAs samples of CAP patients.

To achieve high sensitivity of pathogen detection, both the pathogenicity-related genes and conserved genes were selected and the corresponding sequences were tiled on RPM-IVDC1. In this study, the combination of highly multiplexed PCR with resequencing array allowed for accurate detection of *S. pneumoniae, K. pneumoniae, H. influenzae*, and *M. pneumoniae*. The comparison between RPM-IVDC1 and established qRT-PCR methods demonstrated a concordance for two of two samples in *K. pneumoniae*, nine of 13 samples in *M. pneumoniae*, 12 of 13 samples in *H. influenzae*, and 37 of 37 samples in *S. pneumoniae*. Among the inconsistent results, one sample that was negative for *M. pneumoniae* by qRT-PCR was detected in microarray analysis and confirmed by mycoplasma culture. The disagreement over one sample in *H. influenzae* and five samples in *M. pneumoniae* identification may have resulted from the differences in sensitivity and specificity of the two methods.

The RPM-IVDC1 assay maintained coverage of a large number of pathogens. Due to this advantage, some uncommon types of *Haemophilus* spp., *Streptococcus* spp., and *Neisseria* spp. were identified. As an example, the targeted sequence of adenylate cyclase (*adk*) gene could be used to identify *H. influenzae* by the sequence alignment, while the targeted sequence of outer membrane protein (*skp*) gene could be used to detect *Haemophilus* spp. at species-level. The specific qRT-PCR confirmed 12 of 14 RPM-positive results for *H. influenzae* which might be the causative agents of CAP (Barnes et al., 1987) and was of particular importance for respiratory infections in children (Nascimento-Carvalho, 2001). *Haemophilus parainfluenzae* and *Haemophilus haemolyticus*, the other two types of *Haemophilus* spp., were commonly considered as commensals colonizing the upper respiratory tract asymptomatically (Mukundan et al., 2007; Murphy et al., 2007). However, recent data suggested that occasional clinical disease might be caused by the two intimately related species of *H. influenzae* (Pillai et al., 2000; Anderson et al., 2012). A similar situation occurred in the detection of *Neisseria* spp. where only species-level identification could be achieved by RPM-IVDC1. Interestingly, the *Neisseria* spp. positive results were mainly found among children aged less than 5 years (9/16). Among the children aged under 2 years, the detection rate was highest (38.5%, 5/13), which was similar to the carriage prevalence of *Neisseria lactamica*, a *N. meningitides* related species but living as harmless commensal (Kristiansen et al., 2012). Although RPM-IVDC1 could not identify these organisms at type-level, the species detection information may be valuable for further analysis in the context of outbreak investigation.

The wide coverage of respiratory pathogens by RPM-IVDC1 assay enabled it an ideal tool for real-world diagnostics and surveillance. Besides the pathogens that were frequently detected in the upper airways (e.g., *S. pneumoniae, H. influenzae*), some well-recognized causes of CAP but rarely found in NPAs were detected by RPM-IVDC1. The detection of these atypical bacteria (e.g., *M. pneumoniae, K. pneumoniae*) might be of great predictive value for diagnosing the etiologic agent, which had an essential role in guiding the development of treatment and prevention strategies. *K. pneumoniae* has been a recognized cause of bacterial pneumonia since its discovery (Ko et al., 2002), but the incidence of community-acquired *Klebsiella* pneumonia has apparently declined in China in recent years (Liu et al., 2006). The detection of *K. pneumoniae* in the upper respiratory tract may help the etiologic diagnosis and thus initiate most appropriate and effective therapy in the context of pneumonia. In this study, a total of two samples were identified as *K. pneumoniae* positive by the alignment of two housekeeping loci sequences, *fumC* and *mdh*, which indicated the possibility of *Klebsiella* pneumonia. The subsequent specific real-time confirmed a *Klebsiella pneumoniae* carbapenemases (KPCs)-positive strain from a 53 years old CAP patient. The combination of RPM-IVDC1 result and antibiotic resistance test would be helpful for clinical therapy.

An additional advantage of RPM-IVDC1 assay was the capability of acquiring the actual sequences of the detected targets. The specificity of RPM-IVDC1 could be improved by the automated alignment of target sequences by TESQ. Of the 39 *ply*-positive samples of which C3 score meeting the cutoff value, a total of two samples were identified as *Streptococcus pseudopneumoniae*, which were confirmed by qRT-PCR (Sistek et al., 2012) (data not shown). The advantage of sequence alignment avoided resulting in false-positive for *S*. *pneumoniae*. As for the *Staphylococcus* spp. detection, a total of 10 samples were detected positive for *mecA* allele, a conserved gene encoded penicillin-binding protein causing methicillin-resistant of *S. aureus* (MRSA). The classical *mecA* gene was not restricted to *S. aureus* but was commonly found in other Staphylococci such as, *S. epidermidis, S. haemolyticus*, and *S. pseudintermedius* (Murakami et al., 1991). Our alignment results showed that these *mecA*-positive samples were *Staphylococcus* spp. positive but non- *S. aureus*. The following *S. aureus* specific qRT-PCR confirmed the detection results of RPM-IVDC1, except for one false-positive sample. Therefore, the access to the actual sequences of the targets and automated alignment performed by TESQ could avoid false-positive and improve the specificity of RPM-IVDC1 array.

Some characteristic distributions of the detected pathogens were found by RPM-IVDC1. *S*. *pneumoniae, H. influenzae*, and *M. pneumoniae* were the most significant pathogens detected among these patients and the detection rate of these pathogens were relatively higher among children below the age of 5 years than other age groups, which were in concordance with other studies (Mundy et al., 1995). The pathogen detection rate was relatively higher in winter than in other seasons, which may result from the cold weather (Young, 1924). With the additional four false-negative samples and one sample confirmed by culture, eight of 14 samples positive for *M. pneumoniae* were found in the age group of two to 14 years and nine of 14 positive results were found among male patients. However, there were no statistically significant correlations between the prevalence of *M. pneumoniae* and age or gender in both symptomatic (*p* = 0.91, *p* = 0.22, respectively) and asymptomatic group (*p* = 0.36, *p* = 0.84, respectively) in the study conducted by Spuesens et al. (2013). The phenomena found in our study may be attributed to the limited sample size. The absence of *S. aureus* and lower detection rate of *M. catarrhalis* than that in other studies (Vaneechoutte et al., 1990; Mundy et al., 1995) may be due to the small sample size, sample quality, and the use of antibiotics prior to sampling.

The RPM-IVDC1 assay provided a sensitive and comprehensive alternative to conventional approaches to the diagnosis and surveillance of respiratory infections. However, some issues must be noted. First, a total of three false-positive results were found among these detections. Of these, two specimens were false-positive for *H. influenzae*, one specimen was false-positive for *Streptococcus* spp. The false-positive results were due to the procedures involving opened PCR plates in this assay, such as multiplex PCR, purification, processing, hybridization, and scanning. The three false positive results were not found after retesting. The handling of unsealed specimens for testing by RPM-IVDC1 was one of the major concerns regarding the assay. The application of decontamination procedures, such as dUTP uracil glycosylase treatment, may be operable in preventing from contamination. Notably, our routine use of the assay after the study period for outbreak investigation has not revealed any contamination.

In addition, when this RPM-IVDC1 assay is applied in pathogen detection for severe respiratory infections or outbreak investigation, carefully considerations must be given. The interpretation of testing results may be complicated due to the commensal colonization of the upper respiratory tract and the difficulty of obtaining a high-quality specimen (Blaschke, 2011). In this study, although RPM-IVDC1 detected *S. pneumoniae* in most samples, it was difficult to distinguish colonization from invasive infection. The quantitative assay might be necessary to accurately identify patients with true pneumococcal disease because most commensal microflora appeared to be present at low titers in the samples. On the other hand, although the RPM assay did not determine the titers of the pathogens detected, a negative nasopharyngeal test might be useful to exclude pneumococcal CAP. The species-level identifications of *Staphylococcus* spp., *Haemophilus* spp., *Streptococcus* spp., and *Neisseria* spp. may be of great significance in outbreak investigation when the colonization could be excluded.

In conclusion, the RPM-IVDC1 assay described here demonstrated capability of simultaneous detection of broad-spectrum respiratory pathogens. The mixing of specific and conserved targets, association with highly multiplexed PCR, as well as acquiring actual targeted sequence improved its sensitivity and specificity while maintaining coverage of a large number of pathogens. When introducing this method in clinical diagnostic for severe respiratory infections or epidemic outbreak investigation, carefully considerations about the results interpretation must be given.

## Disclaimers

The views expressed in this article are those of the authors and do not necessarily represent the views of the Chinese Center for Disease Control and Prevention.

### Conflict of interest statement

The authors declare that the research was conducted in the absence of any commercial or financial relationships that could be construed as a potential conflict of interest.
