# Supplementary material for: Association of targeted multiplex PCR with resequencing microarray for the detection of multiple respiratory pathogens
Source: Front Microbiol. 2015 May 28;6:532. doi: 10.3389/fmicb.2015.00532 (PMC4446546; doi:10.3389/fmicb.2015.00532)
Supplement: Supplementary file 1 [file Table1.DOC]

Supplemental Table1 List of PCR primers in primer mix A used for multiplex PCR

| Primer name | organism | gene target | amplicon size (bp) |
| --- | --- | --- | --- |
| ADB1_E1A | Adenovirus B | *E1A* | 555 |
| ADB2_E1A | Adenovirus B | *E1A* | 637 |
| ADC_E1A | Adenovirus C | *E1A* | 373 |
| ADDA_E1A | Adenovirus D | *E1A* | 363 |
| ADDB_E1A | Adenovirus D | *E1A* | 406 |
| ADE_E1A | Adenovirus E | *E1A* | 439 |
| 229E_Spike | Coronavirus 229E | *spike glycoprotein* | 483 |
| HKU1_NP | Coronavirus HKU1 | *nucleocapsid phosphoprotein* | 619 |
| OC43_Spike | Coronavirus OC43 | *spike glycoprotein* | 561 |
| NL63_Spike | Coronavirus NL63 | *spike glycoprotein* | 600 |
| NL63_NP | Coronavirus NL63 | *nucleocapsid protein* | 527 |
| RSVA_N | human respiratory syncytial virus A | *N* | 538 |
| RSVA_G | human respiratory syncytial virus A | *G* | 800 |
| RSVB_N | human respiratory syncytial virus B | *N* | 546 |
| MPVA_M | human metapneumovirus A | *matrix protein* | 354 |
| MPVB_M | human metapneumovirus B | *matrix protein* | 400 |
| MPVB_N | human metapneumovirus B | *nucleoprotein* | 353 |
| PIV1_M | human parainfluenza virus 1 | *matrix protein* | 589 |
| PIV2_HN | human parainfluenza virus 2 | *hemagglutinin-neuraminidase* | 616 |
| PIV3_M | human parainfluenza virus 3 | *matrix protein* | 496 |
| PIV4_HN | human parainfluenza virus 4 | *hemagglutinin-neuraminidase* | 569 |
| PIV4_M | human parainfluenza virus 4 | *matrix protein* | 340 |
| Bocavirus_NP | human bocavirus | *NP1 protein* | 376 |
| Bocavirus_VP | human bocavirus | *VP1 protein* | 411 |
| FluA_M | influenza A virus | *matrix protein* | 916 |
| H1N1_M | influenza A virus (H1N1) | *matrix protein* | 429 |
| H3N2_M | influenza A virus (H3N2) | *matrix protein* | 637 |
| H5N1_M | influenza A virus (H5N1) | *matrix protein* | 345 |
| FluB_M | influenza B virus | *matrix protein 1and BM2 protein* | 633 |
| FluC_HE | influenza C virus | *hemagglutinin-esterase* | 628 |
| FluC_M | influenza C virus | *matrix protein 1* | 637 |
| RV29_5’UTR | human rhinovirus 29 | *5’ untranslated region* | 414 |
| RV93_5’UTR | human rhinovirus 93 | *5’ untranslated region* | 354 |
| RV25_5’UTR | human rhinovirus 25 | *5’ untranslated region* | 490 |
| EV_5’UTR-1 | human enterovirus | *5’ untranslated region* | 782 |
| EV_5’UTR-2 | human enterovirus | *5’ untranslated region* | 647 |
| EV_5’UTR-3 | human enterovirus | *5’ untranslated region* | 770 |
| Arth_TIM-1 | *Arabidopsis thaliana* | *triosephosphate isomerase* | 384 |

Supplemental Table2 List of PCR primers in primer mix B used for multiplex PCR

| Primer name | organism | gene target | amplicon size (bp) |
| --- | --- | --- | --- |
| H1N1_HA1-1 | influenza A virus (H1N1) | *hemagglutinin* | 1585 |
| H1N1_HA1-2 | influenza A virus (H1N1) | *hemagglutinin* | 1310 |
| H1N1_NA1-1 | influenza A virus (H1N1) | *neuraminidase* | 1245 |
| H1N1_NA1-2 | influenza A virus (H1N1) | *neuraminidase* | 804 |
| H3N2_HA3 | influenza A virus (H3N2) | *hemagglutinin* | 1726 |
| H3N2_NA2 | influenza A virus (H3N2) | *neuraminidase* | 1314 |
| H3N2_M | influenza A virus (H3N2) | *matrix protein* | 430 |
| H5N1_HA5 | influenza A virus (H5N1) | *hemagglutinin* | 1642 |
| HA7-1 | influenza A virus | *hemagglutinin 7* | 1558 |
| HA7-2 | influenza A virus | *hemagglutinin 7* | 947 |
| HA7-3 | influenza A virus | *hemagglutinin 7* | 982 |
| NA3-1 | influenza A virus | *neuraminidase 3* | 805 |
| NA3-2 | influenza A virus | *neuraminidase 3* | 1192 |
| H7N7_HA7 | influenza A virus (H7N7) | *hemagglutinin 7* | 897 |
| H7N7_NA7 | influenza A virus (H7N7) | *neuraminidase 7* | 1174 |
| RV15_5’UTR | human rhinovirus 15 | *5’ untranslated region* | 350 |
| RV39_5’UTR | human rhinovirus 39 | *5’ untranslated region* | 456 |
| RV56_5’UTR | human rhinovirus 56 | *5’ untranslated region* | 485 |
| RV85_5’UTR-1 | human rhinovirus 85 | *5’ untranslated region* | 491 |
| RV85_5’UTR-2 | human rhinovirus 85 | *5’ untranslated region* | 452 |
| RV14_5’UTR | human rhinovirus 14 | *5’ untranslated region* | 367 |
| RV70_5’UTR | human rhinovirus 70 | *5’ untranslated region* | 470 |
| RV24_5’UTR | human rhinovirus 24 | *5’ untranslated region* | 557 |
| RV26_5’UTR | human rhinovirus 26 | *5’ untranslated region* | 513 |
| OC43_NP | Coronavirus OC43 | *nucleocapsid protein* | 415 |
| PIV1_HN | human parainfluenza virus 1 | *hemagglutinin-neuraminidase* | 409 |
| PIV2_M | human parainfluenza virus 2 | *matrix protein* | 556 |
| PIV3_HN | human parainfluenza virus 3 | *hemagglutinin-neuraminidase* | 554 |
| B ant_rpoB | *Bacillus anthracis* | *RNA polymerase beta subunit* | 444 |
| B ant_pagA | *Bacillus anthracis* | *protective antigen* | 567 |
| Y pes_gyrA | *Yersinia pestis* | *DNA gyrase subunit A* | 429 |
| Y pes_caf1 | *Yersinia pestis* | *F1 capsule antigen* | 350 |
| M tub_pncA | *Mycobacterium tuberculosis* | *pyrazinamidase* | 362 |
| M tub_embB | *Mycobacterium tuberculosis* | *putative arabinosyltransferase* | 407 |
| M tub_gyrB | *Mycobacterium tuberculosis* | *DNA gyrase subunit B* | 368 |
| Arth_NAC1-1 | *Arabidopsis thaliana* | *NAC1* | 410 |

Supplemental Table3 List of PCR primers in primer mix C used for multiplex PCR

| Primer name | organism | gene target | amplicon size (bp) |
| --- | --- | --- | --- |
| CoxsackieA2_5’UTR | coxsackievirus A2 | *5’ untranslated region* | 345 |
| CoxsackieA4_5’UTR | coxsackievirus A4 | *5’ untranslated region* | 441 |
| CoxsackieA5_5’UTR | coxsackievirus A5 | *5’ untranslated region* | 595 |
| CoxsackieA6_5’UTR | coxsackievirus A6 | *5’ untranslated region* | 325 |
| CoxsackieA8_5’UTR | coxsackievirus A8 | *5’ untranslated region* | 589 |
| CoxsackieA10_5’UTR | coxsackievirus A10 | *5’ untranslated region* | 481 |
| CoxsackieA18_5’UTR | coxsackievirus A18 | *5’ untranslated region* | 456 |
| CoxsackieA21_5’UTR | coxsackievirus A21 | *5’ untranslated region* | 496 |
| CoxsackieA22_5’UTR-1 | coxsackievirus A22 | *5’ untranslated region* | 468 |
| CoxsackieA22_5’UTR-2 | coxsackievirus A22 | *5’ untranslated region* | 414 |
| CoxsackieB2_5’UTR | coxsackievirus B2 | *5’ untranslated region* | 494 |
| CoxsackieB5_5’UTR-1 | coxsackievirus B5 | *5’ untranslated region* | 315 |
| CoxsackieB5_5’UTR-2 | coxsackievirus B5 | *5’ untranslated region* | 594 |
| Echo4_5’UTR | echovirus 4 | *5’ untranslated region* | 338 |
| Echo8_5’UTR | echovirus 8 | *5’ untranslated region* | 548 |
| Echo9_5’UTR | echovirus 9 | *5’ untranslated region* | 384 |
| Echo11_5’UTR-1 | echovirus 11 | *5’ untranslated region* | 432 |
| Echo11_5’UTR-2 | echovirus 11 | *5’ untranslated region* | 344 |
| Echo20_5’UTR | echovirus 20 | *5’ untranslated region* | 496 |
| Echo21_5’UTR | echovirus 21 | *5’ untranslated region* | 526 |
| Echo24_5’UTR | echovirus 24 | *5’ untranslated region* | 508 |
| Echo25_5’UTR | echovirus 25 | *5’ untranslated region* | 498 |
| Echo73_5’UTR | echovirus 73 | *5’ untranslated region* | 553 |
| Polio1_5’UTR | poliovirus 1 | *5’ untranslated region* | 503 |
| Polio2_5’UTR | poliovirus 2 | *5’ untranslated region* | 379 |
| Polio3_5’UTR | poliovirus 3 | *5’ untranslated region* | 534 |
| EV68_5’UTR | enterovirus 68 | *5’ untranslated region* | 333 |
| EV70_5’UTR | enterovirus 70 | *5’ untranslated region* | 512 |
| EV71_5’UTR-1 | enterovirus 71 | *5’ untranslated region* | 471 |
| EV71_5’UTR-2 | enterovirus 71 | *5’ untranslated region* | 490 |
| EV71_5’UTR-3 | enterovirus 71 | *5’ untranslated region* | 490 |
| EV71_5’UTR-4 | enterovirus 71 | *5’ untranslated region* | 432 |
| EV71_5’UTR-5 | enterovirus 71 | *5’ untranslated region* | 461 |
| EV94_5’UTR | enterovirus 94 | *5’ untranslated region* | 488 |
| Arth_NAC1-2 | *Arabidopsis thaliana* | *NAC1* | 376 |

Supplemental Table4 List of PCR primers in primer mix D used for multiplex PCR

| Primer name | organism | gene target | amplicon size (bp) |
| --- | --- | --- | --- |
| Parvo_VP1 | human parvovirus B19 | *VP1 protein* | 538 |
| Parvo_NS | human parvovirus B19 | *non-structural protein* | 573 |
| HHV1_TK | human herpesvirus 1 | *thymidine kinase* | 601 |
| HHV2_TK | human herpesvirus 2 | *thymidine kinase* | 569 |
| HHV3_TK | human herpesvirus 3 | *thymidine kinase* | 618 |
| HHV4_EBNA-1 | human herpesvirus 4 | *EBNA-1 protein* | 385 |
| HHV5_Interleukin | human herpesvirus 5 | *interleukin 10* | 471 |
| HHV6_Chemokine-1 | human herpesvirus 6B | *chemokine* | 342 |
| HHV6_Chemokine-2 | human herpesvirus 6A | *chemokine* | 340 |
| KI_VP1 | KI polyomavirus | *VP1 protein* | 552 |
| WU_VP1 | WU polyomavirus | *VP1 protein* | 602 |
| SARS_NP | SARS coronavirus | *nucleocapsid protein* | 563 |
| SARS_Spike | SARS coronavirus | *spike glycoprotein* | 598 |
| Toro_HE | bovine torovirus | *hemagglutinin-esterase protein* | 467 |
| Toro_NC | human torovirus | *nucleocapsid protein* | 418 |
| Parecho_5’UTR | human parechovirus | *5’ untranslated region* | 577 |
| Aichi_5’UTR | aichi virus | *5’ untranslated region* | 593 |
| Aichi_VP1 | aichi virus | *capsid protein (VP1)* | 394 |
| Measles_HA | measles virus | *hemagglutinin protein* | 404 |
| Measles_M | measles virus | *matrix protein* | 844 |
| Sendai_HN | sendai virus | *hemagglutinin-neuraminidase* | 553 |
| Sendai_M | sendai virus | *matrix protein* | 411 |
| Mumps_HN-1 | mumps virus | *hemagglutinin-neuraminidase* | 343 |
| Mumps_HN-2 | mumps virus | *hemagglutinin-neuraminidase* | 512 |
| Mumps_M | mumps virus | *matrix protein* | 524 |
| Rubella_E1-1 | rubella virus | *structural protein E1* | 471 |
| Rubella_E1-2 | rubella virus | *structural protein E1* | 433 |
| Rubella_NS-1 | rubella virus | *non- structural polyprotein* | 593 |
| Rubella_NS-2 | rubella virus | *non- structural polyprotein* | 645 |
| Pox_crmB | monkeypox/cowpox virus | *tumor necrosis factor receptor II*  *homolog (crmB)* | 541 |
| Pox_HA | monkeypox/cowpox virus | *hemagglutinin* | 534 |
| 229E_NP | Coronavirus 229E | *nucleoprotein* | 494 |
| HKU1_Spike-1 | Coronavirus HKU1 | *spike glycoprotein* | 381 |
| HKU1_Spike-2 | Coronavirus HKU1 | *spike glycoprotein* | 445 |
| RSVB_G | human respiratory syncytial virus B | *attachment glycoprotein* | 459 |
| Arth_NAC1-3 | *Arabidopsis thaliana* | *NAC1* | 352 |

Supplemental Table5 List of PCR primers in primer mix E used for multiplex PCR

| Primer name | organism | gene target | amplicon size (bp) |
| --- | --- | --- | --- |
| BP-prn | *Bordetella pertussis* | *pertactin* | 480 |
| BP-ptxS1 | *Bordetella pertussis* | *pertussis toxin S1 subunit* | 355 |
| CT-gyrA | *Chlamydia trachomatis* | *DNA gyrase subunit A* | 598 |
| CT-incA | *Chlamydia trachomatis* | *inclusion membrane protein A* | 427 |
| CPN-pmpE | *Chlamydophila pneumoniae* | *polymorphic outer membrane protein* | 386 |
| CPS-ompA | *Chlamydophila psittaci* | *major outer membrane protein A* | 503 |
| CD-tox | *Corynebacterium diphtheriae* | *diphtheria tox* | 513 |
| CD-dtxR | *Corynebacterium diphtheriae* | *diphtheria tox repressor* | 445 |
| HI-skp | *Haemophilus influenzae* | *outer membrane protein 26* | 424 |
| HI-adk | *Haemophilus influenzae* | *adenylate kinase* | 356 |
| KP-fumC | *Klebsiella pneumoniae* | *fumarase C* | 389 |
| KP-mdh | *Klebsiella pneumoniae* | *malate dehydrogenase* | 595 |
| LP-mip | *Legionella pneumophila* | *macrophage infectivity potentiator* | 370 |
| LP-gyrB1 | *Legionella pneumophila* | *DNA gyrase subunitB* | 440 |
| LP-gyrB2 | *Legionella pneumophila* | *DNA gyrase subunit B* | 384 |
| MC-mcaP | *Moraxella catarrhalis* | *McaP* | 376 |
| MC-copB | *Moraxella catarrhalis* | *outer membrane protein B2* | 422 |
| MP-adhP1 | *Mycoplasma pneumoniae* | *cytadhesin protein P1* | 481 |
| MP-CARDS | *Mycoplasma pneumoniae* | *CARDS toxin* | 425 |
| PA-gyrB | *Pseudomonas aeruginosa* | *DNA gyrase subunit B* | 610 |
| PA-toxA | *Pseudomonas aeruginosa* | *exotoxin A* | 469 |
| SAU-aroE | *Staphylococcus aureus* | *shikimate dehydrogenase* | 337 |
| SAU-gyrB | *Staphylococcus aureus* | *DNA gyrase subunit B* | 590 |
| SAU-mecA | *Staphylococcus aureus* | *penicillin binding protein* | 490 |
| SAU-luksPV | *Staphylococcus aureus* | *Panton-Valentine leukocidin S* | 586 |
| SP-gyrA | *Streptococcus pneumoniae* | *DNA gyrase subunit A* | 552 |
| SP-ply | *Streptococcus pneumoniae* | *pneumolysin* | 607 |
| GBS-cspA | *Streptococcus agalactiae* | *serine protease* | 402 |
| GBS-gyrB | *Streptococcus agalactiae* | *DNA gyrase subunit B* | 411 |
| GAS-gki | *Streptococcus pyogenes* | *glucose kinase* | 401 |
| GAS-speB | *Streptococcus pyogenes* | *pyrogenic exotoxin B* | 505 |
| NM-porB | *Neisseria meningitidis* | *porin* | 519 |
| NG-porB | *Neisseria gonorrhoeae* | *porin* | 589 |
| NS-gyrA | *Neisseria* spp. | *DNA gyrase subunit A* | 409 |
| FT-fopA | *Francisella tularensis* | *outer membrane protein* | 445 |
| FT-galE | *Francisella tularensis* | *galactose epimerase* | 520 |
| Arth_TIM-2 | *Arabidopsis thaliana* | *triosephosphate isomerase* | 471 |
